# Insulin-Sensitizing Effects of Omega-3 Fatty Acids: Lost in Translation?

**DOI:** 10.3390/nu8060329

**Published:** 2016-06-01

**Authors:** Antigoni Z. Lalia, Ian R. Lanza

**Affiliations:** Division of Endocrinology, Diabetes, Nutrition and Metabolism, Mayo Clinic College of Medicine, 200 First St SW, Rochester, MN 55905, USA; lalia.antigoni@mayo.edu

**Keywords:** insulin resistance, EPA, DHA, *n*-3 PUFA, mitochondria, muscle

## Abstract

Omega-3 polyunsaturated fatty acids (*n*-3 PUFA) of marine origin, eicosapentaenoic acid (EPA), and docosahexaenoic acid (DHA), have been long studied for their therapeutic potential in the context of type 2 diabetes, insulin resistance, and glucose homeostasis. Glaring discordance between observations in animal and human studies precludes, to date, any practical application of *n*-3 PUFA as nutritional therapeutics against insulin resistance in humans. Our objective in this review is to summarize current knowledge and provide an up-to-date commentary on the therapeutic value of EPA and DHA supplementation for improving insulin sensitivity in humans. We also sought to discuss potential mechanisms of *n*-3 PUFA action in target tissues, in specific skeletal muscle, based on our recent work, as well as in liver and adipose tissue. We conducted a literature search to include all preclinical and clinical studies performed within the last two years and to comment on representative studies published earlier. Recent studies support a growing consensus that there are beneficial effects of *n*-3 PUFA on insulin sensitivity in rodents. Observational studies in humans are encouraging, however, the vast majority of human intervention studies fail to demonstrate the benefit of *n*-3 PUFA in type 2 diabetes or insulin-resistant non-diabetic people. Nevertheless, there are still several unanswered questions regarding the potential impact of *n*-3 PUFA on metabolic function in humans.

## 1. Introduction

There is tremendous interest in the health benefits of omega-3 polyunsaturated fatty acids (*n*-3 PUFA), which include the essential fatty acid α-linolenic acid (ALA) and longer-chain fatty acids, eicosapentaenoic (EPA) and docosahexaenoic (DHA), derived from marine organisms. There is an extensive body of literature dedicated to understanding, among the many chronic diseases that may benefit from dietary intake of *n*-3 PUFA, its therapeutic potential in the context of type 2 diabetes. Albeit the FDA supports the use of *n*-3 PUFA as a treatment for hypertriglyceridemia, and the Mediterranean diet as a preventive strategy for cardiovascular disease [1], there is not a clear consensus from human trials on the systematic use of *n*-3 PUFA supplements for people with insulin resistance or type 2 diabetes. Substantial inconsistencies exist between studies of humans compared to rodents. While most studies in rodents suggest a favorable effect of omega-3 fatty acids on glucose utilization and insulin sensitivity, human studies have been conflicting. Although some report improvement in insulin sensitivity with fish oil consumption, the majority of human studies do not recapitulate the findings. Prior review reports extensively covered this subject [2,3,4,5], however, a synopsis of more current knowledge is needed with greater attention to the reasons behind this apparent lack of translatability from rodents to humans. We, therefore, conducted a literature search of recently published studies within the last two years and extended the discussion to include representative studies published earlier. This review focuses on the influence of EPA and DHA on insulin sensitivity and summarizes the outcomes of recent animal studies, human observational studies, and randomized clinical trials, while rendering further insight into mechanistic data on skeletal muscle metabolism and mitochondrial response to *n*-3 PUFA supplementation.

### 1.1. Insulin Resistance

Insulin resistance (IR) is an early metabolic abnormality in the course of obesity, metabolic syndrome, and type 2 diabetes. The prevalence of insulin resistance is high worldwide, with approximately 35% of US adults having insulin resistance [6]. Insulin stimulates skeletal muscle glucose uptake, and inhibits hepatic glucose production and adipose tissue lipolysis. In conditions of insulin resistance, these actions are impaired, leading to a vicious cycle of fasting or postprandial hyperglycemia, elevated free fatty acids, hyperinsulinemia, and pancreatic β-cell dysfunction [7]. Although IR is more evident in obesity [8,9], it is also noted in lean individuals with a high genetic component [10,11,12], and has been related to a constellation of abnormalities, such as skeletal muscle mitochondrial dysfunction [13], ectopic lipid accumulation [14], liver steatosis [15], inflammation [16], oxidative stress [17], and aging [18]. The major culprit, however, for the high prevalence of insulin resistance is a positive energy balance, derived from an excessive food consumption of low nutritional value and a sedentary lifestyle [19,20].

### 1.2. n-3 PUFA

EPA and DHA are very long chain polyunsaturated fatty acids (VLC *n*-3 PUFA) that incorporate into cell membranes following consumption of fatty fish, such as salmon, herring, mackerel, tuna, and sardines. Their biosynthesis is limited in the human body by relatively inefficient desaturase and elongase enzymes that convert ALA into VLC *n*-3 PUFA. Established properties of *n*-3 PUFA are their anti-inflammatory action and lowering effect on triglycerides [21], and they have been used as complimentary strategies in coronary heart disease [22,23] and retinopathy [24], while there is extensive research on improving cognitive disorders [25], telomeric aging [26], and cancer progression [27]. Much controversy still exists on their effect on glucose metabolism and insulin action. Mechanistic information about their action is still under investigation, however, their pleiotropic effects are purported to be related to their chemical structure. The long tail of double bonds confers some unique chemical and physical properties; they are extremely flexible molecules with rapid transitions between a large number of conformers, indicating a key property for entering into the binding pockets of proteins [28]. In addition, their cis bonds limit the ability of the fatty acids to be closely packed when incorporated into cell membranes, driving the formation of lipid domains, such as lipid rafts [29], which then enable or inhibit the interaction with signaling proteins and regulation of downstream pathways [29,30,31]. Thus *n*-3 PUFA are believed to affect membrane fluidity and also modulate expression of genes via regulation of transcription factors related to energy supply, cell cycle regulation, and cell differentiation [32].

## 2. Preclinical Studies of *n*-3 PUFA and Insulin Resistance

### 2.1. High Calorie Diet-Induced Insulin Resistance

There are a plethora of studies on mouse and rat models of insulin resistance following a high carbohydrate (HCD) or high fat (HFD) diet. Rodents fed a HCD or HFD increase their weight, serum, and liver triglycerides and become hyperinsulinemic with impaired glucose tolerance. In a recently published study in rats, HCD enriched with dietary DHA and EPA for 30 days could reverse these symptoms [33]. In this study, fish oil was supplemented as 2%, 5% and 7% of the fat intake. Interestingly, 2% supplementation was not effective in reversing insulin resistance, while 5% and 7% attenuated the HOMA-IR index and had a dose-dependent effect on increasing the expression of genes related to hepatic lipid β-oxidation and decreasing the expression of genes related to hepatic *de novo* lipogenesis (SREBP-1c, ChREBP). This indicates enhancement of insulin action in the liver and a protective effect of *n*-3 PUFA in the course of development of insulin resistance. Nevertheless, the effective doses of *n*-3 PUFA were beyond the recommended levels of the American Heart Association for adult humans, which range up to 4 g/day (~4.5% of fat intake) [34].

In support of the aforementioned study, we recently showed that HFD-induced insulin resistance in mice was partially prevented by substituting 3.4% kcal of saturated fat intake with *n*-3 PUFA [35]. Mice were fed a control diet (10% fat), HFD (60% fat,) or HFD plus EPA/DHA for 10 weeks. Body weight and fat mass increased similarly in HFD and HFD plus EPA/DHA groups, indicating that the protective effects of *n*-3 PUFA were not mediated by modifying adiposity; it has been reported however that *n*-3 PUFA supplementation might ameliorate body weight [36,37]. Following an oral glucose tolerance test, the EPA/DHA group maintained glucose levels similar to controls. Considering that the insulin response at 15 min was similarly increased for both HFD and *n*-3 PUFA, albeit measurements were not done for the whole 2-h duration, the beneficial effect in glycemia is likely to be attributable to greater insulin sensitivity. This is in consistency with other studies reporting greater insulin sensitivity with fish oil supplementation in a HFD background in mice [36,38,39] and rats [39].

In terms of insulin signaling and gene expression, we also showed that *n*-3 PUFA increased the mRNA expression of insulin-stimulated glucose transporter-4 (GLUT4), insulin receptor substrate-1 (IRS1) and glycogen synthase-1 (GYS1) in skeletal muscle, corroborating the finding of enhanced glucose utilization [35]. Consistent with these data, in the same model of HFD-fed mice, Lamping KG *et al.* demonstrated the superiority of fish oil compared to monounsaturated olive oil and *n*-6-enriched fish oil, in restoring basal glucose levels, glucose tolerance, and insulin signaling, including AKT phosphorylation [40]. Similarly in rats, Lionetti *et al.* showed that a HFD rich in fish oil (40% fat) compared to a HFD rich in lard (40% fat) for six weeks, also increased GLUT-4 and IRS1 transcripts expression in muscle, concomitant with improvements in insulin sensitivity, thus yielding some mechanistic explanation of the *n*-3 PUFA effect on skeletal muscle. The rescue of insulin signaling by *n*-3 PUFA, which is initially suppressed by a HFD, has been suggested to be tissue specific, with differential effects on muscle, liver, and adipose tissue [40]. Most studies, however, confirm the beneficial effect of *n*-3 PUFA on muscle, involving insulin receptor (IR) density, IR and IRS1 phosphorylation, phosphatidyloinositol (PI) 3′-kinase activity, and GLUT-4 content [41]. Luo *et al.* also suggested a beneficial effect of DHA in HFD-fed mice on adipose tissue angiogenesis and insulin resistance, via the silent information regulator 1 (SIRT 1) pathway. SIRT1 belongs in a family of proteins which are purported to be involved in the regulation of glucose homeostasis and attenuate insulin resistance via reducing mitochondrial dysfunction [42].

We therefore conclude that observations recapitulated in recent and numerous prior studies [43,44,45,46,47] provide strong evidence that *n*-3 PUFA prevent the reduction in glucose tolerance and insulin sensitivity induced by a high fat diet background in rodents.

### 2.2. Muscle-Liver Glucoregulatory Axis

White P.J. *et al.* proposed a new model of omega-3 action mediated by its active metabolites, termed specialized proresolving mediators (SPM), which include resolvins (Rv), protectins (PD), and maresins (MaR). These bioactive lipid metabolites are, in general, associated with the resolution of obesity-linked inflammation and insulin resistance in high-fat fed mice [48]. The authors suggested that protectin DX (PDX), produced via lipoxygenation of DHA, is responsible for activating a myokine–liver glucoregulatory axis, through stimulation of IL-6 release from skeletal muscle. IL-6 is thought to regulate hepatic glucose production via induction of STAT3 phosphorylation which in turn suppresses expression of gluconeogenic genes in the liver, including peroxisome proliferator-activated receptor γ coactivator-1α (PPARGCo1a), phosphoenolpyruvate carboxykinase (Pck1), and glucose-6-phosphatase (G6pc). Insulin sensitivity was assessed by a hyperinsulinemic-euglycemic clamp with a paired ‘lipid infusion plus PDX or vehicle’, and ‘saline infusion plus PDX or vehicle’. Interestingly PDX improved both peripheral and hepatic insulin action in WT mice, while results were abrogated in IL-6 null mice. In diabetic db/db mice, PDX lowered glycemia without affecting insulin concentration, indicating the superiority of its effect on hepatic insulin action inhibiting gluconeogenesis [49]. PDX also displayed higher phosphorylation of the AMP-activated protein kinase (AMPK) in muscle, which is considered as another potent mechanism mediating the effects of omega-3 fatty acids on insulin sensitivity [50]. This study suggested that the therapeutic benefits of *n*-3 PUFA might be due, in part, to the distinct actions of their bioactive metabolites, which may further mediate cross-organ communication.

### 2.3. Anti-Inflammatory Effects

Chronic macrophage-mediated inflammation is considered a hallmark of insulin resistance, and omega-3 fatty acids are purported to exhibit anti-inflammatory effects. A landmark study by Oh *et al.* reported that the G-protein-coupled receptor 120 (GPR120) expressed predominantly in mature adipocytes, macrophages, and hepatic stellate cells, functions as an omega-3 fatty acid receptor/sensor. By signaling through GPR 120, *n*-3 PUFA inhibit both TLR and TNF-α inflammatory signaling pathways and likely mediate M1–M2 macrophage polarization by decreasing the expression of inflammatory genes (IL-6, TNF-a, MCP-1, IL-1b, iNOS, CD11c) and increasing the expression of anti-inflammatory genes in adipose tissue (IL-10, MGL1, YM-1, Clec7a, MMR). GPR120 KO mice were glucose intolerant, hyperinsulinemic, and displayed skeletal muscle and hepatic insulin resistance, which was not ameliorated by *n*-3 PUFA supplementation. On the contrary, HFD-insulin-resistant WT mice supplemented with *n*-3 PUFA improved overall insulin sensitivity through mitigation of inflammation and increased adipose tissue glucose uptake [47]. Other studies also explored the prevention or reversal of IR in HFD-induced obese mice via modulation of adipose tissue inflammation, with a reported increase in anti-inflammatory cytokines (adiponectin) in plasma [51].

### 2.4. Hepatoprotection

Additional liver lipidomic profiling by Oh *et al*. revealed that *n*-3 PUFA ameliorated HFD-induced steatosis, supporting the view that omega-3 treatment can reverse non-alcoholic fatty liver disease (NAFLD) mediated in large part by GPR 120 [47]. Hepatoprotection and prevention of NAFLD, with repression of hepatic stellate cell activation and fibrogenesis in the liver, was also reported in mice fed HFD-enriched with fish oil for six weeks [39] and 12 weeks [52]. This was observed in parallel with a decrease in liver oxidative stress and was associated with improvements in HOMA-IR, adiponectin plasma levels, and overall insulin sensitivity. Liu X *et al*. also confirmed that EPA supplementation alone was efficacious in suppressing body fat accumulation and alleviated insulin resistance measured by OGTT in a HFD, HCD background. EPA alone also efficiently alleviated hepatic steatosis by modulating the suppression of adipocytokines (adiponectin) and inflammatory cytokines (TNFa, IL-6), and suppressed SREBP-1c-mediated lipogenesis while enhancing lipid β-oxidation (Liu, Xue *et al.* 2013). In line with these observations, a recent review by Delarue *et al*. concluded that LC-*n*-3 PUFA decrease liver steatosis, but do not reverse already established histologic features of non-alcoholic stehatohepatitis (NASH) [53].

A study by Poudyal *et al*. measured the independent effects of ALA, EPA, and DHA in high calorie-fed rats and all *n*-3 PUFA individually reduced inflammation in both heart and liver, as well as reducing cardiac fibrosis and hepatic steatosis. These effects were associated with complete suppression of stearoyl-CoA desaturase 1 (Scd-1) activity, a marker implicated in cardiovascular disease, insulin resistance, and obesity [37].

### 2.5. Oxidative Stress

Amelioration of oxidative stress in various tissues was also reported in a spontaneously hypertensive obese rat model (SHROB) of the metabolic syndrome. Treatment with EPA/DHA for 13 weeks increased the activity of antioxidant enzymes in erythrocytes, abdominal fat, and kidneys, and lowered the plasma C-reactive protein (CRP) inflammation marker. Of note, the magnitude of the activation varied depending on different EPA/DHA ratios, with 1:2 having the strongest effect on oxidative stress *versus* 1:1 and 2:1 [54]. In contrast, we did not find any effect of fish oil on skeletal muscle antioxidant enzymes activity from HFD-mice, including sodium dismutase (SOD1) and catalase, although catalase mRNA expression was found to be significantly higher in the fish oil-supplemented group [35].

### 2.6. n-6:n-3 PUFA Ratio

A study demonstrating the anti-inflammatory properties of *n*-3 PUFA via suppression of toll-like receptor 4 (TLR4) activation in muscle reported improvements in inflammatory markers (TNFα, CRP, IL-6) and insulin resistance as measured from oral glucose and insulin tolerance tests (OGTT and ITT) [55]. These effects, however, were determined by a specific ratio of *n*-6:*n*-3 PUFA of 1:1, and were not replicated by a ratio of 4:1, suggesting that a balance among polyunsaturated fatty acids might be a more important contributor to metabolic health, rather than absolute levels of *n*-3 PUFA, as reported previously [56,57]. Other studies in non-rodent animals also attempted to delineate the metabolic importance of the *n*-6:*n*-3 PUFA ratio. Duan *et al.* fed pigs with either a 1:1, 2.5:1, 5:1, and 10:1 *n*-6:*n*-3 PUFA ratio and showed that the 5:1 ratio led to the highest growth performance, while the 1:1 diet led to increased muscle mass and lowest adipose tissue mass, with concomitant changes in inflammatory markers [58]. Although the metabolic significance of the *n*-6:*n*-3 PUFA ratio is still under investigation, considering the very high average ratio of the Western diet, 16–17:1, it is likely that an optimized *n*-6:*n*-3 PUFA ratio may exert beneficial effects on lipid metabolism, inflammation status, and other metabolic pathways.

### 2.7. n-3 PUFA Role in Aging

Aging is purported to be associated with a higher inflammatory status and insulin resistance. We, therefore, fed young and old mice with normal chow enriched with either EPA or DHA and performed large-scale proteomics and mRNA sequencing analysis from muscle tissue samples. Among the top metabolic pathways affected by EPA and DHA, was downregulation of the acute phase response signaling pathway, suggesting that *n*-3 PUFA ameliorated the inflammatory status in the muscle of old mice compared to young controls [32]. In addition, EPA supplementation enhanced muscle protein quality in the old mice by reducing carbamylation, a post-translational modification known to be driven by inflammation [32,59]. Further, we detected no differences in glucose tolerance or insulin sensitivity, indicating that *n*-3 PUFA might be beneficial only when a certain level of metabolic dysfunction is established, but not when insulin sensitivity is within a normal range. Aging is also associated with reduced mitochondrial function, which is implicated in the pathogenesis of insulin resistance [13]. Discussion of the effect of *n*-3 PUFA on mitochondrial function in the context of aging is reported under the section, “EPA and DHA Effect on Skeletal Muscle Metabolism”.

### 2.8. Preclinical Studies in Primates

The majority of reports in rodents suggest beneficial effects of VLC-*n*-3-PUFA on insulin sensitivity and glucose tolerance; however, data in humans are ambiguous. An interesting report comes from a study in non-human primates, male rhesus monkeys, which develop features of the metabolic syndrome under a HCD. Supplementation with 4 g/day EPA + DHA for six months prevented fructose-induced hypertriglyceridemia and insulin resistance, as assessed by intravenous glucose tolerance testing. The area under the curve (AUC) for glucose remained fairly stable, however, both groups exhibited increased insulin concentrations, suggesting β-cell compensation for insulin resistance. Fish oil mitigated the hyperinsulinemia, as well as the increase in leptin and apolipoprotein E, without any changes in adiponectin, body weight, and fat mass [60]. Since primates provide a better model of human metabolism than rodents, these observations bring further interest in the translational value of the findings.

## 3. Human Studies of *n*-3 PUFA and Insulin Resistance

### 3.1. Observational Studies in Adults

Most observational studies assess the relationship of omega-3 fatty acids levels, measured in plasma and erythrocyte membranes, with the prevalence of metabolic syndrome (MetS). Due to the nature of the cross-sectional studies and the large cohorts of participants involved, the most common and feasible method used to assess insulin resistance is the homeostasis model assessment (HOMA-IR), derived from fasting glucose and insulin concentrations. Consequently, HOMA-IR is not a representative measurement of insulin sensitivity in postprandial conditions and could lead to erroneous assumptions. The majority of observational studies reflect a favorable effect of *n*-3 PUFA on insulin action. Most of the studies found an inverse association between *n*-3 PUFA content and the index of insulin resistance [61,62,63], even in populations with a high intake of fish oil and a low risk for MetS and diabetes, like the Alaska Eskimos [64,65], indicating that *n*-3 PUFA could assist in the prevention or treatment of insulin resistance in humans. Of note, the associations were significant even after adjusting for age, gender, BMI, and ethnicity [62]. Biosynthesis of EPA in the body measured by the Δ5 desaturase index was associated with insulin resistance in normoglycemic, but not in glucose intolerant, adults in a Cree Canadian population [63]. In a Korean population, *n*-3 PUFA were not predictors of increased risk for metabolic syndrome [66]. Nevertheless, in a prospective study in the same population of Korean healthy adults, ages 40–69 years, who were followed for three years for MetS and CVD outcomes, the consumption of fish oil was associated with a lower risk for MetS among men, but not among women [67]. In a recently performed observational study from the National Heart, Lung, and Blood Institute (NHLBI) Family Heart study, in 4941 participants, there was no association of fish oil consumption with MetS in a large U.S. population [68]. Consecutively, there appear to be geographic differences in the responsiveness to *n*-3 PUFA, potentially related to environmental factors and/or genetic variations, as well as usual dietary habits and lifestyles.

### 3.2. Observational Studies in Children and Adolescents

Observational studies in children render similar results to studies in adults, showing an inverse association of omega-3 fatty acids with HOMA-IR [69,70], lower plasma levels of EPA in obese children with IR compared with obese children without IR [70], and beneficial associations between *n*-3 PUFA and lipid profile [71]. Conflicting results come from a Danish study, where DHA was associated with a poor metabolic profile [72]. However, in obese children, DHA content had an inverse association with BMI [73], suggesting that overall dietary management, including omega-3 fatty acids, could be an important tool in managing childhood obesity.

## 4. *n*-3 PUFA in Human Clinical Trials

### 4.1. Meta-Analyses

A summary of recent RCTs is shown on Table 1. There was no change in insulin sensitivity in 10 out of 13 identified randomized, double-blind, placebo-controlled trials. In consistency, a systematic meta-analysis (of 11 RCTs published until October 2010, with *n* = 618 participants) concluded that *n*-3 PUFA consumption did not affect insulin sensitivity [74]. However in a sensitivity analysis by measures of IS, a positive relationship was found in the HOMA sub-group compared to the control, but not in the QUICKI sub-group. This observation is difficult to interpret, because HOMA and QUICKI are equivalent and crude measurements of IS, yet it cannot be disregarded. Another systematic review, which examined the effect of EPA and DHA on metabolic syndrome risk factors, concluded that there are no clear effects on metabolic syndrome markers, except for an improvement in blood pressure and the well-established hypotriglyceridemic effect. Interestingly, lower doses of *n*-3 PUFA were associated with further benefits of reducing pro-atherogenic small dense particles (sdLDL), whereas greater doses (≥3 g/day) were associated with increases in LDL cholesterol [75].

### 4.2. Limitations in Translating Results from Animal to Human Studies

As it will be discussed in the following sections, human intervention studies, in their majority, have failed to recapitulate the protective effect of EPA and DHA on glucose metabolism and insulin sensitivity that have been observed in rodents. The reasons for this incongruence between humans and animals, besides the conceivable genetic and phenotypic interspecies differences, are unclear. However, the majority of studies in rodents have been preventive, whereas human studies examine the curative potential of omega-3 fatty acids to reverse an already established metabolic dysfunction. This methodological discordance is compounded by the use of relatively crude indices of insulin sensitivity, including QUICKI or HOMA-IR, compared to more sensitive tools such as OGTT, mixed-meal challenge, and the hyperinsulinemic-euglycemic clamp. Furthermore, there is a huge variety in the dosage and duration of *n*-3 PUFA administered in both animal and human studies, precluding easily unified conclusions (Table 2). With the majority of human studies being observational, and a paucity of randomized, double-blinded, placebo-controlled trials of adequate size and power, any conclusions for the systematic use of *n*-3 PUFA in regulating human metabolic health await further verification.

### 4.3. Representative RCTs

In order to conclusively evaluate the long-term effects of *n*-3 PUFA on insulin sensitivity in humans, it is essential to conduct carefully controlled studies with sufficient duration of supplementation and gold-standard measurements of outcomes. While most intervention studies vary from one to three months, our team recently conducted a six-month randomized, double blind, placebo-controlled trial in 31 insulin-resistant and overweight or obese adults who received 3.9 g/day EPA+DHA [76]. This was considered an adequate timeframe for the long-term effects of a pharmacologically relevant dose of *n*-3 PUFA. The study was carefully conducted with the use of a pancreatic hyperinsulinemic-euglycemic clamp and, consistent with previous reports, there was no effect of *n*-3 PUFA on whole body insulin sensitivity in the fasted postabsorptive state. We also observed no changes in postprandial glucose disposal and insulin secretion following a mixed-meal challenge. Furthermore, plasma inflammatory markers did not change with the intervention in consistency with a few other studies [76,77,78]. There is one more RCT by Derosa *et al.*, with a robust study design of 3 g/day *n*-3 PUFA supplementation for six months and use of a hyperinsulinemic-euglycemic clamp to measure insulin sensitivity. The study included a large cohort of patients with combined dyslipidemia (control group *n* = 79, treatment group *n* = 78), who also received an oral fat load to assess inflammation response. The results demonstrated that, although there was an improvement in insulin sensitivity in the *n*-3 PUFA group, the effect was not significantly different from the controls. In addition, participants received a controlled-energy diet with 600 Kcal daily energy deficit, and were encouraged to increase their physical activity, both of which might have confounded the actual *n*-3 PUFA effect. In this metabolically unhealthy cohort, *n*-3 PUFA resulted in an improved response to oral fat load with higher HDL levels, and lower triglycerides, inflammatory markers (CRP, TNFa, IL-6), cell adhesion molecules, and other atherosclerotic risk factors, including metalloproteinases 2 and 9 [79].

Further, two double-blind, placebo-controlled RCTs, which used the intravenous glucose tolerance test (IVGTT) to assess insulin sensitivity following a three-month intervention with an adequate dose of *n*-3 PUFA, also reported no change in insulin sensitivity (S_I_). Giacco *et al.* randomly assigned isoenergetic diets rich in monounsaturated (MUFA) or saturated fat (SFA) to 162 healthy Caucasian adults and further subdivided the groups to receive 3.6 g/day fish oil, or a placebo. None of the four diets affected insulin sensitivity (S_I_) and the insulin disposition index or BMI. When the investigators accounted for higher (>4.85) or lower (<4.85) *n*-6:*n*-3 PUFA ratio, the same results persisted [86]. The second study by Spencer *et al.* focused on the effect of *n*-3 PUFA on adipose tissue in adults with impaired glucose tolerance, impaired fasting glucose, or metabolic syndrome. Supplementation with 4 g/day EPA and DHA reduced adipose (but not muscle) macrophages and plasma macrophage chemoattractant protein 1 (MCP-1) and increased capillary density. Despite these favorable effects on indices of chronic inflammation, there was no change in SI [78].

Two additional large RCTs of six-month *n*-3 PUFA supplementation showed no change in insulin sensitivity based on HOMA-IR [83,87]. In specific, a cohort of 124 elderly Iranian men received 300 mg/day of EPA and DHA, or a placebo, and showed reduced serum triglycerides, however, no other effects were detected in fasting blood sample variables and the index of insulin resistance [83]. Of note, this was a relatively small dose of *n*-3 PUFA, and albeit the time frame was adequate to achieve a cumulative effect in lowering triglycerides, it was less likely to have a prominent effect on insulin sensitivity compared to other studies where a larger dose of *n*-3 PUFA was used. The OPTILIP study in UK provided five diets of different *n*-6:*n*-3 PUFA ratios in 258 middle-aged and older adults who were at risk of ischemic heart disease. The aim of the study was to identify an optimal ratio for dietary recommendations to reduce cardiovascular risk. Therefore it included a food-based intervention rather than use of supplements, posing some limitations in standardizing the actual *n*-3 PUFA intake. The ratios varied from 10:1 (controls) to 5:1, 3:1 (EPA, DHA, ALA), 3:1 (ALA), and 3:1 (EPA, DHA). Decreasing the *n*-6:*n*-3 PUFA did not influence measures of insulin resistance, but confirmed the favorable effect in reducing triglycerides and proatherogenic sdLDL. Consecutively, the aforementioned studies provided evidence which does not support a long-term beneficial effect of *n*-3 PUFA in insulin sensitivity, despite measurable effects on cardiovascular risk factors and inflammation.

### 4.4. n-3 PUFA Effect and Inflammation

Inflammation is purported to be a leading mechanism in insulin resistance and metabolic disease. While most of the RCTs did not demonstrate a favorable effect of *n*-3 PUFA on insulin sensitivity, there are studies which reported improvement, as measured by HOMA-IR, and these studies had as a common denominator cohorts of participants with a high background inflammatory status. Browning *et al.*, using a cross over study design, divided 30 overweight or obese women into tertiles of inflammatory status based on concentrations of sialic acid. Following 4.2 g/day of three-month *n*-3 PUFA supplementation and an oral glucose tolerance test, women in the top tertile for inflammation demonstrated improved insulin sensitivity, whereas those at the reference lowest tertile did not [85]. The women in the raised inflammatory status also had higher BMI and insulin resistance, as well as fibrinogen and CRP. However, the fact that the improvement in insulin sensitivity was not accompanied by significant changes in CRP, TNF-α, IL-6, α-1 anti-chymotrypsin, plasminogen activator inhibitor-1 (PAI-1), and α-1 acid glycoprotein (AGP), is suggestive that these plasma inflammatory markers are crude measurements of the *n*-3 PUFA anti-inflammatory effect or that the effect is mediated via other pathophysiological pathways. This corroborates the null findings from RCTs on relatively healthy adults with either moderate hypertriglyceridemia (Skulas-Ray, 2011 #5679) [95], or chronic lifestyle stress (Muldoon, 2016 #5670) [96] and low dietary EPA and DHA intake. Despite the beneficial effect on triglycerides, low, moderate, or pharmacological doses of *n*-3 PUFA had no effect on serum inflammatory markers (CRP and IL-6) *versus* a placebo.

Interesting data were also reported from patients with chronic renal failure under hemodialysis, where systemic inflammation and nutritional deficiency of proteins and essential fatty acids are prevalent and predictive of poor clinical outcomes. Supplementation of 2.4 g/day for two months led to significant improvement in HOMA-IR, ferritin levels, and inflammatory markers of TNF-α, IL-6 and hs-CRP with no significant changes in anthropometric characteristics [97]. In a second cross-sectional study of 111 hemodialysis patients, adequate intake of *n*-3 PUFA and a lower ratio of *n*-6:*n*-3 PUFA were associated with a higher total skeletal muscle mass [98]. Since skeletal muscle is the main target of insulin action, this study is relevant to understanding the effect of *n*-3 PUFA on insulin sensitivity and underscores the potential anabolic properties of *n*-3 PUFA in humans with an increased inflammatory status. Similarly, in patients with cancer cachexia from pancreatic cancer, it is well recognized that proinflammatory cytokines, such as TNF-α and IL-6, can induce the cachectic state, and acute-phase protein inflammatory response strongly predicts poor prognosis. Twenty-six patients with a median age of 56 years received an escalated high dose of 6 g/day EPA, which reversed the rate of weight change from a median loss of 2 kg/month to a median gain of 0.5 kg/month. This significant weight stabilization was achieved during the first month of the intervention and maintained for the remaining two months. The effect was also associated with the downregulation of proinflammatory cytokines, although other mechanisms at the level of gene expression and transcription cannot be excluded [99].

Another sensitive population group who could benefit from *n*-3 PUFA intervention are women with polycystic ovary syndrome (PCOS). PCOS is a common endocrinology disorder associated with obesity, a high degree of insulin resistance, and increased risk factors for diabetes and cardiovascular disease. An intervention in young Iranian PCOS women for two months of 1.2 g/day *n*-3 PUFA led to 21.8% improvement in HOMA-IR, compared to a placebo. This was in parallel with significant increase in serum adiponectin, an adipose tissue-derived cytokine with anti-atherogenic and anti-inflammatory effects. In addition, there were favorable effects in the lipid profile, with improvement in total and LDL cholesterol, but no significant changes in HDL or CRP [80].

The regulation of adipokine secretion by *n*-3 PUFA, including adiponectin and leptin, has been previously reviewed [100] and verified in recent studies [93]. A compelling study comes from Yamamoto *et al.*, who used EPA administration preoperatively for one month in patients undergoing cardiac surgery, a procedure liable to initiate an acute stress inflammatory response. Although they did not find any changes in insulin sensitivity or cardiac adverse events, pre-EPA treatment significantly decreased the neutrophil–lymphocytre ratio (NLR), mediated possibly by increases in adiponectin levels. This resulted in a decreased risk for post-operative infection through enhanced cell-mediated immunity, and underscored a whole new field for novel applications of *n*-3 PUFA in the clinical setting.

In this regard, formation of specialized pro-resolving mediators (SPM) from *n*-3 PUFA, termed resolvins, protectins, and maresins, may underlie some of the beneficial effects attributed to their precursors, EPA and DHA. Inflammatory response as a protective mechanism should be self-limited. However, in conditions where inflammation persists (acute phase response or chronic), SPM have the intriguing ability to selectively stimulate resolution of the inflammation without immune suppresion. Consequtively SPMs may be very effective from a therapeutic standpoint by terminating neutrophil recruitment and inflammatory cytokines release, and stimulating macrophage clearance and tissue regeneration [101,102]. Although there is an emerging body of evidence on their anti-inflammatory action and organ protective effects, as identified in the eye, kidney, lung, and periodontal tissue [103,104], clinical studies in humans to address the potential therapeutic benefits of SPMs in insulin-sensitive tissues and in the context of insulin resistance and glucose tolerance are needed.

### 4.5. n-3 PUFA and Energy-Restricted Diets

Improvement in insulin sensitivity as measured by HOMA-IR was also noted in conditions of hypocaloric diets [90,91], which suggested that fish oil exerts positive effects on fasting insulin, and this was reported to be independent of weight loss or changes in plasma triacylglycerol and adiponectin concentrations [90]. Of note, these beneficial effects were seen with relatively low doses of *n*-3 PUFA (Table 1). There still remains skepticism about whether the effects of omega-3 fatty acids were confounded by the robust insulin-sensitizing effects of caloric restriction [105], or whether there is a positive interaction of *n*-3 PUFA with dietary and exercise modifications of energy intake and expenditure.

In this regard, *n*-3 PUFA have also been studied for their efficacy in weight-loss interventions. This is particularly relevant because adiposity is a great determinant of insulin resistance [106]. Nevertheless, results have not been unanimous among human studies, with previous reports showing reduced body fat and increased resting fat oxidation in healthy adults [107], and reduced trunk fat and adipocyte diameter in type 2 diabetes patients, without changes in insulin sensitivity as measured by an insulin clamp [108]. When coupled with energy restricted diets, there was no effect of *n*-3 PUFA on body fat of young athletes [109] or overweight women [110], but there was a report of greater weight loss and waist circumference reduction in overweight men [111]. When fish oil was added to exercise regimens, there was an independent effect on body fat reduction in overweight and obese adults [112], but no effect in lean young male volunteers [113]. Also, in severely obese women, aerobic exercise plus a very low-calorie diet plus 2.8 g/day *n*-3 PUFA, led to a greater reduction in BMI and hip circumference compared to exercise plus diet plus placebo. Although these studies did not assess insulin sensitivity, they could indicate a potential beneficial role of *n*-3 PUFA via reducing adiposity. The modest reduction in body fat or adipocyte size in cohorts of obese adults is not in contrast with their aforementioned anabolic effect on skeletal muscle, since previous reports have been supportive of a tissue-specific action of *n*-3 PUFA.

### 4.6. RCTs in Children

Clinical trials in children and adolescents with insulin resistance and obesity also rendered favorable outcomes, with general improvements in fasting glucose, insulin, triglycerides, BMI [114], HOMA-IR, TNFa, leptin, adiponectin [115], and blood pressure [116]. Nevertheless, more accurate measurements of insulin sensitivity in children and adolescent populations are missing and would be required in order to establish the therapeutic benefit of *n*-3 PUFA in this vulnerable population.

### 4.7. n-3 PUFA in Type 2 Diabetes

A brief overview of randomized clinical trials in type 2 diabetes patients is summarized in Table 3. It has been long proposed that omega-3 fatty acids do not provide beneficial effects on the glycemic control of patients with established type 2 diabetes [2,117]. Two meta-analyses, including 18 and 23 RCTs respectively, with large numbers of participants, were concordant in outcomes, describing a decrease in triglycerides, a potential increase in LDL, and no effect on glycemic control or fasting insulin from *n*-3 PUFA supplementation [2,117].

In addition, meta-analyses of prospective studies had variable outcomes and reported no effect of *n*-3 PUFA on diabetes risk [126,127], beneficial effects in Asian populations [127,128,129], no associations among Europeans, and increased risk for incidence of diabetes in U.S. populations [128]. The observed differences between geographical regions could be a reflection of heterogeneous diets, including fish consumption, as well as racial and ethnic genetic differences. A more recent observational study by Lou DJ *et al.* [130] assessed the relationship of serum *n*-3 PUFA levels with IR and non-alcoholic fatty liver disease (NAFLD) in patients with type 2 diabetes, and reported that *n*-3 PUFA levels were significantly lower in T2DM and NAFLD and negatively correlated with HOMA-IR.

Among the most recent RCTs (Table 3), only one reported an improvement in HOMA-IR, with a concomitant decrease in NEFA following supplementation of 4 g/day for 2.5 months [118]. The majority of the studies which used the hyperinsulinemic-euglycemic clamp to assess insulin sensitivity, showed no beneficial effect of omega-3 fatty acids irrespective of duration and dosage [108,120,123,125]. There were, however, two randomized, double-blind, placebo-controlled studies by Mostad IL *et al.* which reported a moderate but significant deterioration of glycemia in subjects treated with fish oil. C-peptide responses also tended to be enhanced by fish oil, while an interesting effect was noticed in increased fat utilization compared to carbohydrate oxidation in the fasting state, following nine weeks of the intervention [121,122].

### 4.8. Preventive Versus Therapeutic Action of n-3 PUFA

Most animal studies are designed to prevent the effects of insulin resistance in a HFD or HCD background, while human studies are designed to reverse already established IR, an important distinction that may explain conflicting outcomes. An interesting approach to this notion was taken by conducting trials of an acute administration of lipid emulsions, enriched with omega-3 fatty acids *versus* placebo. Acute lipid infusions showed improvement in insulin sensitivity measured by a six-hour hyperinsulinemic-euglycemic clamp in six healthy young men [92]. In Type 2 DM patients, however, Mostad *et al.* used a four-hour lipid infusion with 0.04 g/kg *n*-3-PUFA enrichment and found no acute effect on insulin sensitivity. Also, neither insulin secretion, nor markers of oxidative stress, leptin, adiponectin, or energy expenditure, measured by indirect calorimetry, were affected [120]. Therefore, more trials are needed to verify the acute action of *n*-3 PUFA in insulin sensitivity and their potential role as preventive strategies for metabolic syndrome in humans.

Positive results were reported from a study with a preventive design by Delarue and colleagues. Insulin resistance was acutely induced in eight healthy, young, lean men with the use of dexamethasone for two days and participants were studied before and after three weeks of 1.8 g/day EPA plus DHA (6/day of fish oil) supplementation. The study demonstrated a 17% decrease in insulinemia as measured by the six-hour AUC following an oral glucose load. There was no change in the rate of glucose appearance and disappearance, substrate oxidation, c-peptide secretion, or endogenous glucose production. The authors suggested that, since insulin secretion and insulin clearance as measured by the c-peptide to insulin molar ratio remained stable, the decrease in insulinemia was indicative of improved peripheral insulin sensitivity [131]. This study corroborated their previous findings in five healthy volunteers who responded with 40% decrease in insulinemia following a CHO load, albeit there was a 6% increase in glycemia [132]. These two studies indicated that fish oil can only partially prevent insulin resistance acutely induced by dexamethasone in humans.

A compelling approach was also introduced by Toktam *et al.* in a cohort of patients with schizophrenia or bipolar disorder treated with second-generation antipsychotics, which are unfavorably related to hyperglycemia and insulin resistance. The study was designed in a randomized double-blind fashion to evaluate the early intervention with omega-3 fatty acids supplementation on insulin resistance, caused by concomitant treatment with olanzapine and a sodium valproate or lithium combination. The study outcome was negative and *n*-3 PUFA did not change HOMA-IR, although there were trends of improvement in fasting insulin; yet no data were generated on postprandial insulin action, which is expected to be impaired with long-term antipsychotic treatment [133]. Of note, the intervention was of short duration, six weeks, with a very low dose of omega-3 fatty acids, less than 1 g/day, and none of the groups exhibited hyperglycemia with the antipsychotic treatment [82], suggesting that further evaluation is warranted in this group of patients.

## 5. EPA and DHA Effect on Skeletal Muscle Metabolism

### 5.1. Skeletal Muscle Lipid Content

Lipotoxicity from ectopic lipid accumulation in skeletal muscle tissue due to conditions of caloric surplus has been purported to contribute to the development of insulin resistance [14,134]. Leading hypotheses, however, do not implicate increased absolute levels of TG, but incomplete lipid β-oxidation and the accumulation of toxic intermediates, including long-chain acylcarnitines (LCACoA) [135], ceramides [136], and diacylglycerols (DAG) [137], which interfere with normal mitochondrial function and insulin signaling [138]. Indeed, in a mouse model of HFD-induced insulin resistance, we showed that muscle triglycerides accumulation was not prevented by fish oil, yet insulin sensitivity improved. This was due to a partial attenuation of LCACoA levels in all muscle fractions of total homogenate, sarcoplasmic and mitochondrial. Also, fish oil attenuated the accumulation of the most abundant ceramide species (palmitic and stearic) [35]. This is consistent with a report of combined treatment with rosiglitazone and *n*-3 PUFA in mice during a HFD, which mitigated the increase in ceramide content, and improved insulin sensitivity [139]. In agreement, Stephens *et al.* demonstrated that acute administration of *n*-3 PUFA in an intravenous lipid emulsion, altering the *n*-6:*n*-3 ratio, did not change total muscle acylcarnitine content; nevertheless, it prevented much of the decline in insulin sensitivity and in pyruvate dehydrogenase complex activity (PDCa), a rate-limiting enzyme in glucose oxidation, which can be allosterically inhibited by *n*-6 PUFA administration [92]. Therefore we conclude that improvements in peripheral insulin sensitivity by *n*-3 PUFA are not necessarily mediated by changes in total lipid content.

### 5.2. Skeletal Muscle Mitochondrial Function

Mitochondrial dysfunction is intertwined with the insulin resistance phenotype, as these organelles are responsible for lipid oxidation and for sustaining the metabolic demands of skeletal muscle. In this regard, we reported that fish oil increased expression of master transcriptional factors of mitochondrial biogenesis, including PGC1a and nuclear respiratory factor 1 (nrf1) [35]. Others also showed that omega-3 fatty acids are ligands for receptors that activate PGC1a [140], leading to an increased expression of PGC1a, mitochondrial transcription factor A (TFAM), cytochrome c oxidase, and mitochondrial membrane potential [141]. These studies indicate that dietary *n*-3 PUFA can prevent or reverse impairments in muscle mitochondria content or function. However, maximal mitochondrial oxidative capacity and phosphorylation efficiency did not change, pointing towards other mechanisms being associated with insulin signaling. This is consistent with reports in insulin-resistant humans, where oxidative capacity did not change after six months of EPA and DHA supplementation, measured *in vitro* from muscle biopsies and verified *in vivo* with magnetic resonance spectroscopy [76]. In light of these observations, Herbst *et al.* showed that EPA and DHA are incorporated into mitochondrial membranes and exert their effect by increasing mitochondrial sensitivity for ADP, and thus increasing submaximal, but not maximal, oxidative capacity [142]. Nevertheless, in the context of aging, where mitochondrial function is known to be impaired [13], EPA, but not DHA, partially improved mitochondrial oxidative capacity in old mice, without stimulating mitochondrial biogenesis or restoring age-related reductions in mitochondrial abundance. The effects were rather mediated by an improvement in mitochondrial protein quality by reducing deleterious post-translational modifications [32]. These findings would suggest that, depending on the underlying dysfunction (lipid overload, insulin resistance, aging) or severity, there could be differential effects of *n*-3 PUFA on skeletal muscle mitochondria function. In the context of aging, there are no available data, to date, on aged humans to support this assumption. However, we have unpublished data on elderly humans who were supplemented with 3.9 g/day of EPA/DHA for four months, which demonstrated that EPA/DHA could not reverse the age-related reduction in mitochondrial oxidative capacity, despite favorable effects on muscle protein homeostasis.

## 6. Conclusions

Despite promising strong outcomes in animal studies, there is no substantial cumulative evidence, to date, that VLC *n*-3 PUFA dietary supplementation can serve as a therapeutic strategy for insulin resistance in humans. Even less promising, are the data in regulating glycemic control in patients with type 2 diabetes. However, there is a strong indication that VLC *n*-3 PUFA may be used as preventive strategies based on their pleiotropic effects and their potential in regulating inflammation and innate immunity at the level of macrophages and the paracrine or endocrine function of cytokines. Although EPA and DHA have been mostly studied together, additional RCTs are needed to delineate their differential and independent effects elicited on muscle, liver, and adipose tissue with regards to insulin action. Further exploration and understanding of these mechanisms could be beneficial for long-term preventive strategies for chronic diseases and for promoting favorable outcomes in the acute clinical setting.

## Figures and Tables

**Table 1:** Summary of human clinical trials in non-diabetic individuals.

NON DIABETIC							
Study	Objective/Participants	Dose	Duration	Method	Effect on Insulin Sensitivity
RCTs double-blinded, placebo-controlled							
Lalia AZ *et al.* 2015 [76]	IR, overweight, n_t_ = 14, n_c_ = 11	3.9 g/day	6 months	Pancreatic Clamp	No change
Root M *et al.* 2013 [77]	Effects of FO on vascular health and arterial stiffness. Overweight BMI>23 kg/m^2^, young adults 18–30 y, n_t_ = 30, n_c_ = 27	1.7 g/day	1 month	FBG	No change
Spencer M *et al.* 2013 [78]	Effect of FO on adipose tissue inflammation, obese, IR with MetS n_t_ = 19, n_c_ = 14	4 g/day	3 months	IVGTT	No change
Derosa G *et al.* 2012 [79]	Dyslipidemic patients n_t_ = 78, n_c_ = 79	3 g/day	6 months	Clamp	No change
Mohammadi E *et al.* 2012 [80]	Iranian, PCOS women, overweight or obese, 20–35 y, n_t_ = 32, n_c_ = 32	1.2 g/day	2 months	HOMA-IR	Improved
Kelly DS *et al.* 2012 [81]	Hypertriglyceridemic men *n* = 14–17/group	3 g/day DHA	3 months	HOMA-IR, Matsuda index	No change
Toktam F *et al.* 2010 [82]	Schizophrenia or bipolar disorder n_t_ = 20, n_c_ = 21	<1 g/day	1.5 months	HOMA-IR	No change
Fakhrzadeh H *et al.* 2010 [83]	Effects of FO on serum lipid profile of elderly Iranians, ≥65 y, *n* = 124	300 mg/day	6 months	HOMA-IR	No change
Ahren B *et al.* 2009 [84]	Young 20–37 y: lean BMI 20–26 kg/m^2^ *n* = 12, obese BMI 29–35 kg/m^2^ *n* = 10. Old 50–65 y: lean *n* = 16, obese *n* = 11 Crossover design	3g + CLA 3 g/day, control 6 g/day	3 months	Mixed meal	No change/Decreased in older obese
Browning LM *et al.* 2007 [85]	Overweight women with inflammatory phenotype. Top quartile *n* = 12, low quartile *n* = 18 Crossover design with 1 month washout.	4.2 g/day	3 months	OGTT	Improved top tertile
Giacco *et al.* 2007 [86]	Healthy men and women, *n* = 162	3.6 g/day	3 months	IVGTT	No change
Griffin MD *et al.* 2006 [87]	Effect of *n*-6:*n*-3 ratio with 4 diets between 5:1 and 3:1, men and postmenopausal women from the OPTILIP study, 45–70 y, *n* = 258	6% Kcals	6 months	HOMA-IR and QUICKI	No change
RCTs singe-blinded, placebo-controlled							
Oh, PC *et al.* 2014 [88]	Hypertriglyceridemia, *n =* 44 in each group	1 g/day, 2 g/day, 4 g/day	2 months	QUICKI	No change
Rajkumar H *et al.* 2014 [89]	Healthy, 40–60 y, overweight, 4 groups: placebo, omega-3 fatty acids, probiotic, omega-3 fatty acids +probiotic. *n =* 15 per group	<1 g/day	1.5 month	HOMA-IR	Improved
Ramel *et al.* 2008 [90]	Effect of energy-restricted diets with FO, *n =* 324, European, young 20–40 y, overweight or obese, 4 diets: no seafood n_c_ = 80, lean fish 150 g cod 3/week *n =* 80, fatty fish 3/week *n =* 84, fish oil EPA/DHA capsules *n =* 80	1.3 g/day	2 months	HOMA-IR	Improved
Soares de Oliveira Carvalho AP *et al.* 2014 [91]	Effect of hypocaloric diet plus FO in women with MetS, 30–45 y, n_t_ = 15, n_c_ = 15	0.41 g/day	3 months	HOMA-IR	Improved
Stephens FB *et al.* 2014 [92]	Healthy young men: saline *vs.* 10% *n*-6 PUFA, *vs.* 2:1 *n*-6:*n*-3 PUFA, *n =* 6	total 20 g	acute IV lipid infusion	Clamp	Improved
RCTs—no placebo							
Yamamoto T *et al.* 2014 [93]	Hyperlipidemic patients, 54–84 y, n_t_ = 31, n_c_ = 29	900 mg/day EPA	3–6 months	HOMA-IR	Improved
Yamamoto T *et al.* 2014 [93]	Patients undergoing cardiac surgery, 54–86 y, n_t_ = 10, n_c_ = 12	1.8 g/day EPA	1 month	HOMA-IR	No change
Tsitouras PD *et al.* 2008 [94]	6 men and 6 women over 60 y	720 g fatty fish/week + 15 mL sardine oil/day	2 months	Octerotide insulin suppression testing	Improved
Meta-analyses of RCTs							
Akinkuolie *et al.* 2011 [74]	11 RCTS	0.138–11 g/day	6 weeks–6 months	HOMA-IR/QUICKI/Clamp/IVGTT	No change

*n*-3 PUFA: omega-3 polyunsaturated fatty acids; *n*-6 PUFA: omega-6 polyunsaturated fatty acids; FO: fish oil; n_t_: number of participants in the treatment group; n_c_: number of participants in the control group; y: years of age; IR: insulin resistance; FBG: fasting blood glucose; IVTT: intravenous tolerance test; HOMA-IR: homeostasis model assessment of insulin resistance.

**Table 2:** Confounders in translating animal–human studies.

	Etiology for Discrepancies in Studies of *n*-3 PUFA Action
1	Dosage of *n*-3 PUFA
2	Ratio EPA:DHA
3	Source of fish oil
4	Absorption and Bioavailability
5	Duration of intervention
6	Type of placebo
7	*n*-6:*n*-3 ratio
8	Type of cohort (young *vs.* old, NG *vs.* IFG *vs.* IR *vs.* DM, lean *vs.* overweight *vs.* obese, race, inflammatory status, comorbidities, usual dietary habits, and lifestyle)
9	Method to assess IS (hyperinsulinemic euglycemic clamp *vs.* HOMA-IR *vs.* OGTT)
10	Preventive study or therapeutic
11	Size and power of study

*n*-3 PUFA: omega-3 polyunsaturated fatty acids; EPA: eicosapentaenoic acid; DHA: docosahexaenoic acid; NG: normal glucose; IFG: impaired fasting glucose; IR: insulin resistance; DM: type 2 diabetes mellitus; HOMA-IR: homeostasis model assessment of insulin resistance; OGTT: oral glucose tolerance test.

**Table 3:** Summary of human studies of the *n*-3 PUFA effect on Type 2 DM patients.

TYPE 2 DM					
Study	Participants	Dose	Duration	Method	Effect on Insulin Sensitivity
RCTs					
Farsi PF *et al.* 2014 [118]	Effect of FO on IS and NEFA, *n =* 44	4 g/day	2.5 months	HOMA-IR/QUICKI	Improved
Crochemore IC 2012 [119]	Effect of FO on IR and lipemia in obese women, *n =* 41, single-blind	2.5 g/day and 1.5 g/day	1 month	HOMA-IR/QUICKI	No change
Mostad IL *et al.* 2009 [120]	*n =* 11, crossover design	0.04 g/kg	acute lipid infusion	Clamp	No change
Mostad IL *et al.* 2008 [121]	Normotriglyceridemic without insulin treatment, n_t_ = 12, n_c_ = 14, placebo-controlled	5.9 g/day	9 weeks	Clamp	Decreased
Kabir M *et al.* 2007 [108]	Effect of FO on adiposity and atherogenic markers in women, *n =* 27	3 g/day	2 months	HOMA-IR/Clamp (in a subgroup of *n =* 5)	No change
Rasic-Milutinovic Z *et al.* 2007 [97]	Hemodialysis patients or chronic renal failure, *n =* 35	2.4 g/day	2 months	HOMA-IR	Improved
Mostad IL *et al.* 2006 [122]	Normotriglyceridemic, *n =* 26, double-blind controlled	5.9 g/day	1 week/9 weeks	Clamp	Decreased
Rivellese AA 1996 [123]	Hypertryglyceridemia, n_t_ = 8, n_c_ = 8, double-blind, placebo-controlled	2.7 g/day for 2 mon then 1.7 g/day for 4 mon	6 months	Clamp	No change
McManus *et al.* 1996 [124]	Well-controlled T2DM, *n =* 11, crossover design	~2.5 g/day	3 months	FSIGT	No change
Annuzi *et al.* 1991 [125]	NIDDM, male, *n =* 8, double-blind crossover design	10 g/day	0.5 month	Clamp	No change
Meta-analyses of RCTs					
Hartweg 2008 [117]	23 RCTs, *n =* 1075	3.5 g/day (mean)	9 weeks (mean)	FBG, FI	No change
Montori 2000 [2]	18 RCTs, *n =* 823	3–18 g/day	12 weeks (mean)	FBG	No change

FO: fish oil; n_t_: number of participants in the treatment group; n_c_: number of participants in the control group; IS: insulin sensitivity; IR: Insulin resistance; NEFA: non esterified fatty acids; FBG: fasting blood glucose; FI: fasting insulin; FSIGT: frequently sampled intravenous glucose tolerance test; HOMA-IR: homeostasis model assessment of insulin resistance.
